# FLT3-ITD transduces autonomous growth signals during its biosynthetic trafficking in acute myelogenous leukemia cells

**DOI:** 10.1038/s41598-021-02221-2

**Published:** 2021-11-22

**Authors:** Kouhei Yamawaki, Isamu Shiina, Takatsugu Murata, Satoru Tateyama, Yutarou Maekawa, Mariko Niwa, Motoyuki Shimonaka, Koji Okamoto, Toshihiro Suzuki, Toshirou Nishida, Ryo Abe, Yuuki Obata

**Affiliations:** 1 Division of Cancer Differentiation, National Cancer Center Research Institute, 5-1-1 Tsukiji, Chuo-ku, Tokyo, 104-0045 Japan; 2 Research Institute for Science & Technology, Tokyo University of Science, Noda, Chiba 278-8510 Japan; 3 Department of Applied Chemistry, Faculty of Science, Tokyo University of Science, Shinjuku-ku, Tokyo, 162-8601 Japan; 4 Department of Chemistry, Faculty of Science, Tokyo University of Science, Shinjuku-ku, Tokyo, 162-8601 Japan; 5 SIRC, Teikyo University, 2-11-1 Kaga, Itabashi-ku, Tokyo, 173-8605 Japan; 6 National Cancer Center Hospital, Tsukiji, Chuo-ku, Tokyo, 104-0045 Japan

**Keywords:** Biochemistry, Cancer, Cell biology

## Abstract

FMS-like tyrosine kinase 3 (FLT3) in hematopoietic cells binds to its ligand at the plasma membrane (PM), then transduces growth signals. *FLT3* gene alterations that lead the kinase to assume its permanently active form, such as *internal tandem duplication (ITD)* and *D835Y* substitution, are found in 30–40% of acute myelogenous leukemia (AML) patients. Thus, drugs for molecular targeting of FLT3 mutants have been developed for the treatment of AML. Several groups have reported that compared with wild-type FLT3 (FLT3-wt), FLT3 mutants are retained in organelles, resulting in low levels of PM localization of the receptor. However, the precise subcellular localization of mutant FLT3 remains unclear, and the relationship between oncogenic signaling and the mislocalization is not completely understood. In this study, we show that in cell lines established from leukemia patients, endogenous FLT3-ITD but not FLT3-wt clearly accumulates in the perinuclear region. Our co-immunofluorescence assays demonstrate that Golgi markers are co-localized with the perinuclear region, indicating that FLT3-ITD mainly localizes to the Golgi region in AML cells. FLT3-ITD biosynthetically traffics to the Golgi apparatus and remains there in a manner dependent on its tyrosine kinase activity. Tyrosine kinase inhibitors, such as quizartinib (AC220) and midostaurin (PKC412), markedly decrease FLT3-ITD retention and increase PM levels of the mutant. FLT3-ITD activates downstream in the endoplasmic reticulum (ER) and the Golgi apparatus during its biosynthetic trafficking. Results of our trafficking inhibitor treatment assays show that FLT3-ITD in the ER activates STAT5, whereas that in the Golgi can cause the activation of AKT and ERK. We provide evidence that FLT3-ITD signals from the early secretory compartments before reaching the PM in AML cells.

## Introduction

FLT3 is a member of the type III receptor type tyrosine kinase (RTK) family and is expressed in the PM of hematopoietic cells^1–3^. Upon stimulation with FLT3 ligand, the receptor undergoes dimerization and autophosphorylates its tyrosine residues, such as Tyr591 and Tyr842^3–5^. Subsequently, it activates downstream molecules, such as AKT, extracellular signal-regulated kinase (ERK), and transcription factors^3,6^. Activation of these cascades results in the growth and differentiation of host cells, leading to normal hematopoiesis^2^. Therefore, gain-of-function mutations of *FLT3* cause autonomous proliferation of myeloid cells, resulting in the development of AML^2,7,8^.

**Figure 1:**
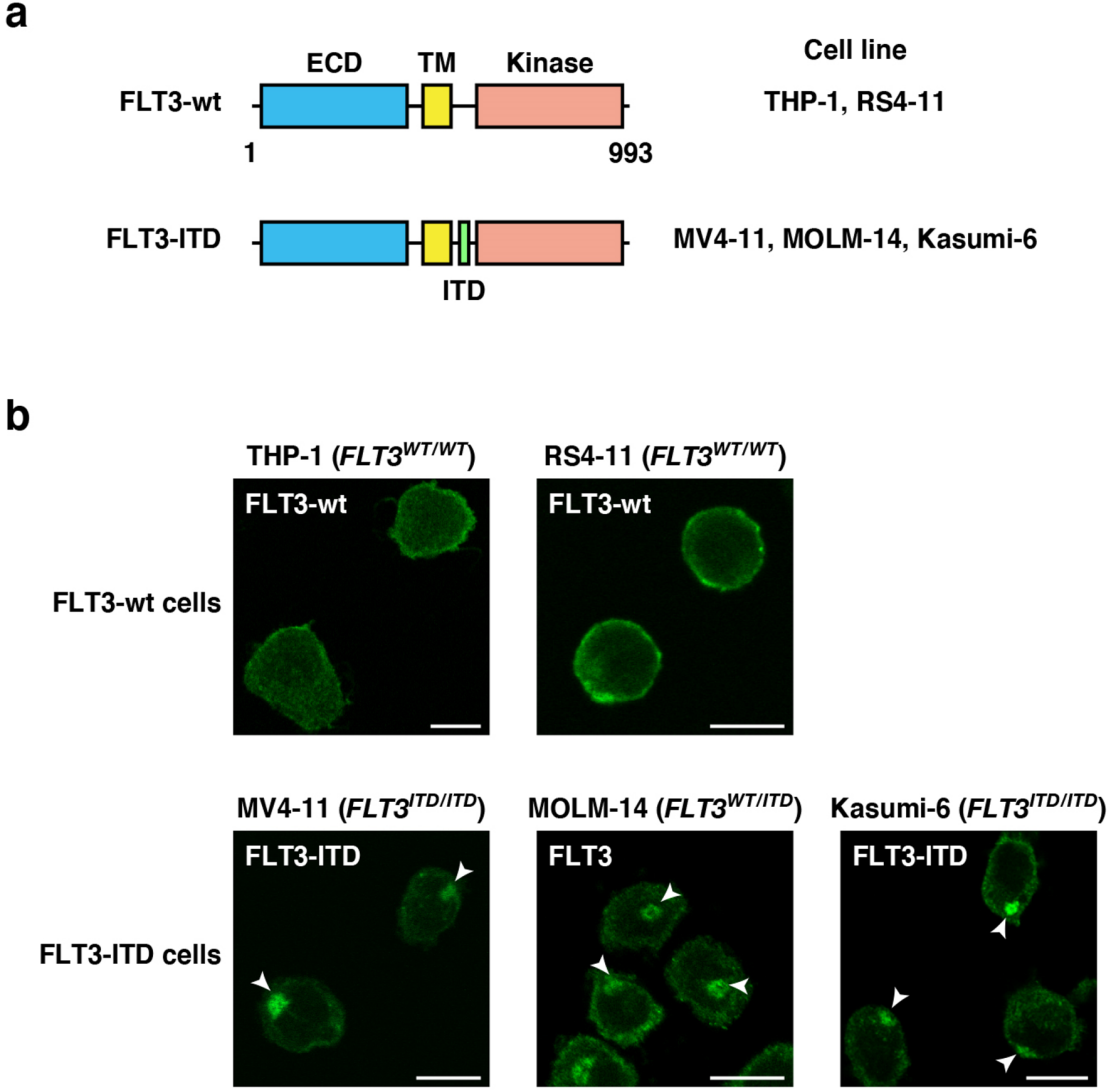
FLT3-ITD mislocalizes to the perinuclear region in AML cells. (**a**) Schematic representations of wild-type FLT3 (FLT3-wt) and an FLT3 internal tandem duplication (FLT3-ITD) mutant showing the extracellular domain (ECD, blue), the transmembrane domain (TM, yellow), the kinase domain (pink), and the ITD (green). (**b**) Fixed THP-1, RS4-11, MV4-11, MOLM-14, or Kasumi-6 cells were permeabilized and subsequently immunostained with anti-FLT3 ECD antibody. Arrowheads indicate the perinuclear region. Bars, 10 µm. Note that FLT3-wt localized to the plasma membrane, whereas FLT3-ITD accumulated in the perinuclear region.

FLT3 is composed of an N-terminal extracellular domain, a transmembrane region, a juxta-membrane (JM) domain, and a C-terminal cytoplasmic tyrosine kinase domain^1,3,6^ (see Fig. 1a). Alterations of the *FLT3* gene that lead the kinase to constitutive activation are seen in 30–40% of AML cases^6,8^. Internal tandem duplication (ITD) into the JM region of FLT3 interferes with its auto-inhibitory ability^9^. In addition, a D835Y substitution in the FLT3 activation loop stabilizes the tyrosine kinase domain in an active state^1,10^. Thus, signal transduction pathways from FLT3 mutants have been investigated^6,11–13^, and molecular targeting drugs for blocking the mutants have been developed for the treatment of AML patients^7,8,14^. Previous studies showed that FLT3-ITD accumulates in the wrong compartments, resulting in low amounts of the mutant in the PM, compared with the allocation of FLT3-wt^4,5,15–17^. Although FLT3-ITD is suggested to activate signal transducers and activators of transcription 5 (STAT5) soon after synthesis^4,18–20^, the precise subcellular localization of the mutant and the relationship between the mislocalization and growth signals remain unclear.

Recently, we reported that KIT, a type III RTK, accumulates in intracellular compartments, such as endosomal/lysosomal membrane and the Golgi apparatus, in mast cell leukemia (MCL), gastrointestinal stromal tumor (GIST), and AML^21–24^. Mutant KIT in leukemia localizes to endosome–lysosome compartments through endocytosis, whereas that in GIST stops in the Golgi region during early secretory trafficking. We further showed that blockade of KIT trafficking to the signal platform inhibits oncogenic signals^24–26^, suggesting that trafficking suppression is a novel strategy for suppression of tyrosine phosphorylation signals.

In this study, we show that endogenous FLT3-ITD aberrantly accumulates in the perinuclear region in AML cells. In our co-staining assays, we found that the perinuclear region was consistent with the Golgi region but not with the ER, endosomes, or lysosomes. The Golgi retention of FLT3-ITD is decreased by tyrosine kinase inhibitors, such as AC220 and PKC412, suggesting that the mutant stays in the Golgi region in a manner that is dependent on its kinase activity. Interestingly, FLT3-ITD can activate AKT and ERK in the Golgi region before reaching the PM. Inhibiting the biosynthetic trafficking of FLT3-ITD from the ER to the Golgi by brefeldin A (BFA) or 2-methylcoprophilinamide (M-COPA) can block the activation of AKT and ERK by FLT3-ITD. We also confirmed that STAT5 is activated by FLT3-ITD in the ER. Our findings provide evidence that FLT3-ITD signaling occurs at intracellular compartments, such as the Golgi apparatus and ER, in AML cells.

## Results

### In human leukemia cells, wild-type FLT3 localizes to the PM, whereas FLT3-ITD accumulates in the perinuclear region

To examine the localization of endogenous FLT3, we performed confocal immunofluorescence microscopic analyses on human acute leukemia cell lines with an anti-FLT3 luminal-faced N-terminal region antibody. For immunostaining, we chemically fixed and permeabilized THP-1 (acute monocytic leukemia, *FLT3*^*WT/WT*^), RS4-11 (acute lymphoblastic leukemia, *FLT3*^*WT/WT*^), MV4-11 (AML, *FLT3*^*ITD/ITD*^), MOLM-14 (AML, *FLT3*^*WT/ITD*^), and Kasumi-6 (AML, *FLT3*^*ITD/ITD*^)^27–30^ (Fig. 1a). In FLT3-wt leukemia cell lines (THP-1 and RS4-11), the wild-type receptor was mainly found at the PM (Fig. 1b, upper panels). In sharp contrast, in *ITD*-harboring AML cells (MV4-11, MOLM-14, and Kasumi-6), the anti-FLT3 antibody particularly stained the perinuclear region (Fig. 1b, lower panels, arrowheads). The anti-FLT3 cytoplasmic domain antibody also stained the perinuclear region in these *ITD*-positive cells (Suppl. Fig. 1), supporting the results of FLT3-ITD mislocalization. Since these three cell lines have different *ITD* sequences^27–29^, the accumulation of FLT3 in the perinuclear region was independent of the inserted amino acid sequences but dependent on *ITD* insertion. These results suggest that *ITD* causes FLT3 retention in the perinuclear compartment in AML cells.

### FLT3-ITD but not FLT3-wt localizes to the perinuclear Golgi region in leukemia cells

**Figure 2:**
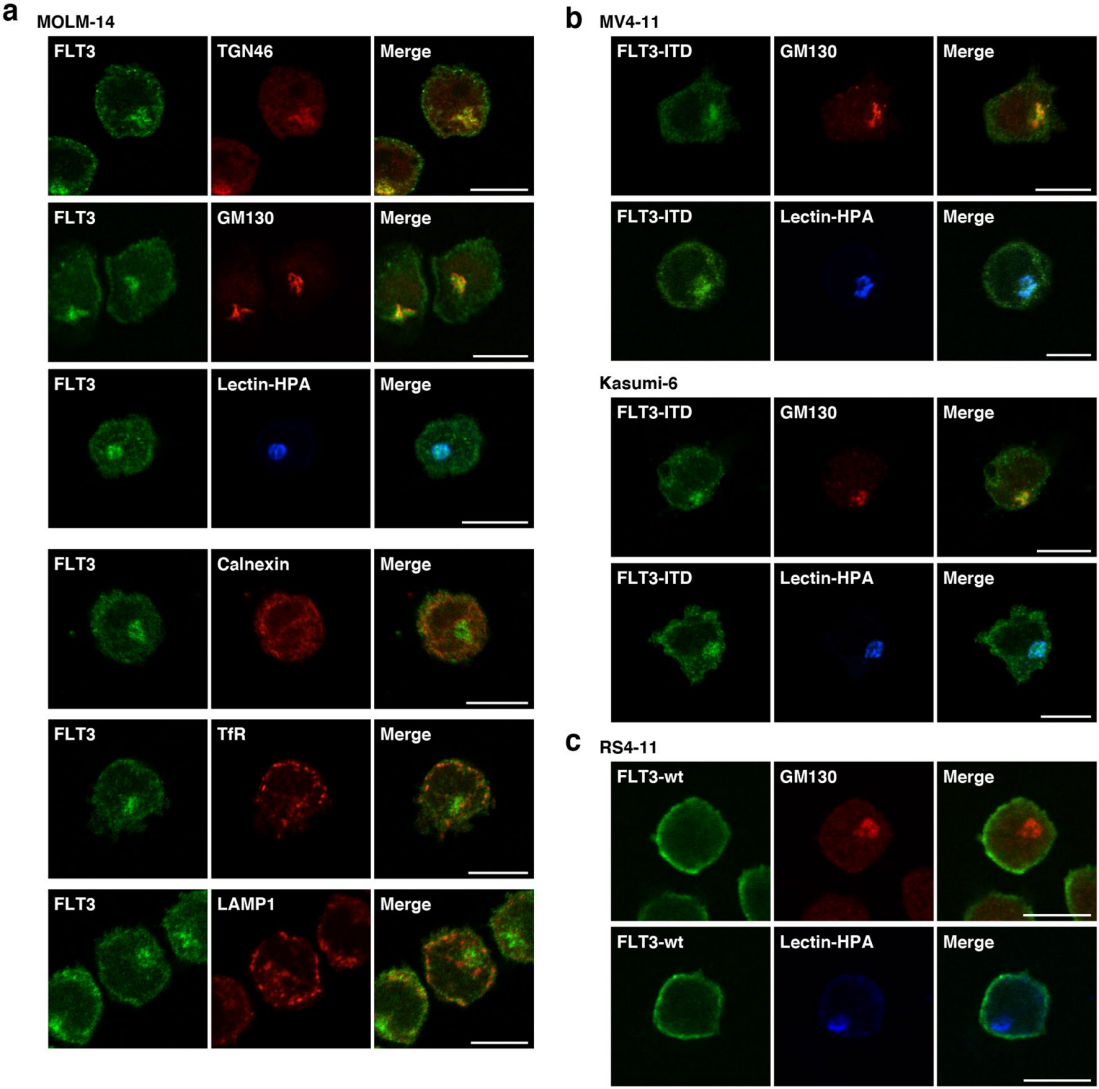
Endogenous FLT3-ITD localizes to the perinuclear Golgi region in AML cells. (**a**–**c**) MOLM-14 (**a**), MV4-11, Kasumi-6 (**b**), or RS4-11 cells (**c**) were stained for FLT3 (green) in conjunction with the indicated organelle markers (red or blue). TGN46 (Golgi marker, red), GM130 (Golgi marker, red); lectin-HPA (Golgi marker, blue); calnexin (ER marker, red); TfR (endosome marker, red); LAMP1 (lysosome marker, red). Bars, 10 µm. Note that FLT3-ITD accumulated in the Golgi region in AML cells.

Next, we investigated the perinuclear region, where FLT3-ITD is found, by examining AML cell lines using co-staining assays. First, we immunostained for FLT3 (green) in conjunction with *trans*-Golgi network protein 46 kDa (TGN46, Golgi marker, red), Golgi matrix protein 130 kDa (GM130, Golgi marker, red), lectin-HPA (Golgi marker, blue), calnexin (ER marker, red), transferrin receptor (TfR, endosome marker, red), or lysosome-associated membrane protein 1 (LAMP1, lysosome marker, red) in MOLM-14 cells. As shown in Fig. 2a, perinuclear FLT3 was co-localized with the Golgi markers but not with the ER marker calnexin. Furthermore, localization of endosomal/lysosomal vesicles was inconsistent with that of perinuclear FLT3 (Fig. 2a), indicating that FLT3-ITD localizes to the Golgi region in MOLM-14 cells. Similar results were obtained from immunofluorescence assays using both MV4-11 and Kasumi-6 cells (Fig. 2b; Suppl. Fig. 2a,b). In these cells, a fraction of FLT3 was found outside the ER region (Fig. 2a; Suppl. Fig. 2a,b), indicating that the receptor could localize in PM of *ITD*-bearing cells. We were unable to find co-localization of FLT3-wt with Golgi markers, such as lectin-HPA and GM130, in RS4-11 cells (Fig. 2c), indicating that *ITD* leads FLT3 to mislocalize to the Golgi region in leukemia cells.

### FLT3-ITD remains at the Golgi region in a manner dependent on its tyrosine kinase activity in AML cells

**Figure 3:**
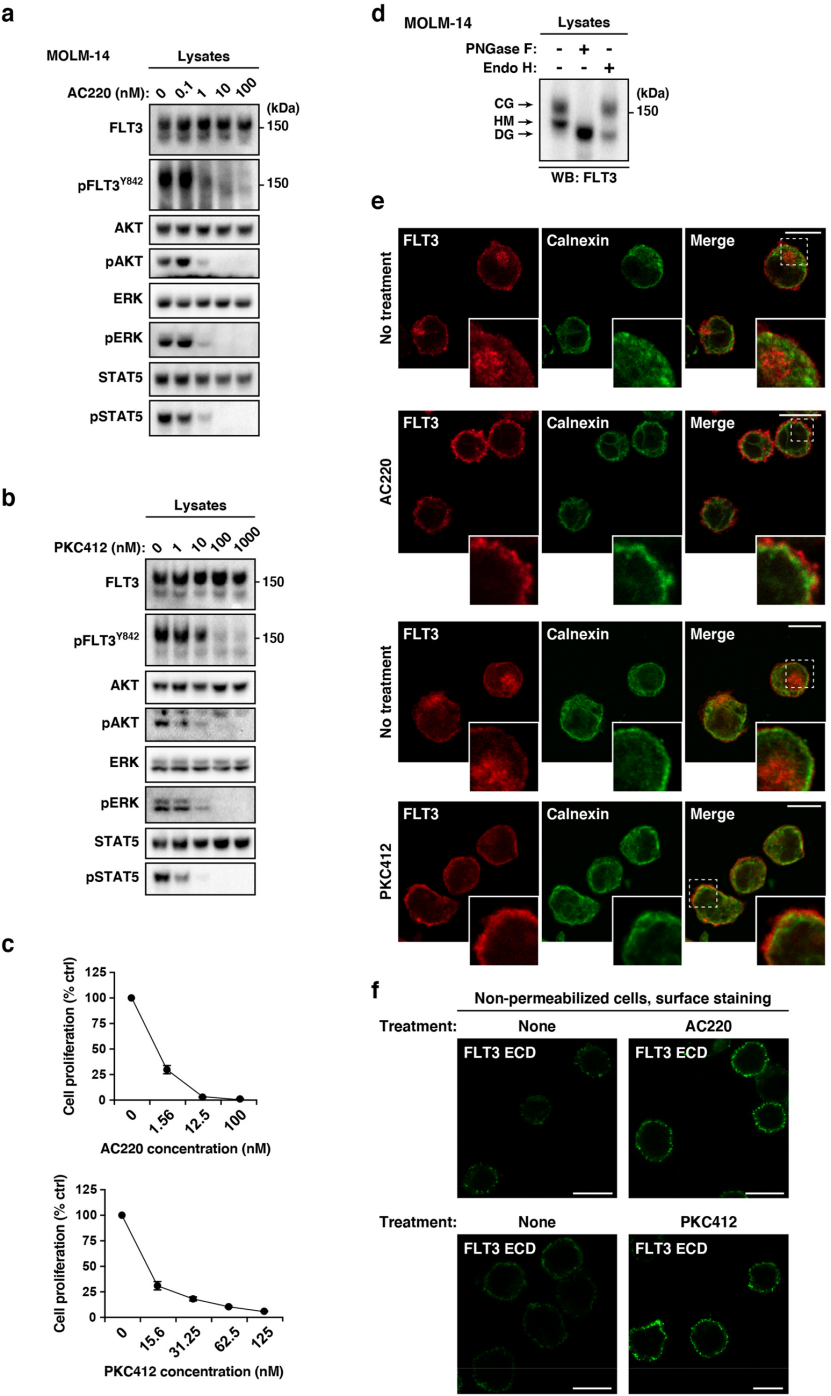
FLT3-ITD retention in the Golgi region is dependent on its tyrosine kinase activity. (**a**, **b**) MOLM-14 cells were treated for 4 h with AC220 (**a**) or PKC412 (**b**). Lysates were immunoblotted for FLT3, phospho-FLT3 Tyr842 (pFLT3^Y842^), AKT, pAKT, ERK, pERK, STAT5, and pSTAT5. Full length blots are presented in Supplementary Fig. 5. (**c**) MOLM-14 cells were treated with AC220 (upper graph) or PKC412 (lower graph) for 48 h. Cell proliferation was assessed by ATP production. Results are means ± *s.d.* (*n* = 3). (**d**) Lysates from MOLM-14 were treated with peptide *N*-glycosidase F (PNGase F) or endoglycosidase H (endo H) then immunoblotted with anti-FLT3 antibody. *CG* complex-glycosylated form, *HM* high mannose form, *DG* deglycosylated form. Full length blots are presented in Supplementary Fig. 5. (**e**, **f**) MOLM-14 cells were treated with 10 nM AC220 or 100 nM PKC412 for 8 h (**e**) or 16 h (**f**). (**e**) Fixed cells were permeabilized, then immunostained with anti-FLT3 (red) and anti-calnexin (ER marker, green). Insets show the magnified images of the boxed area. Bars, 10 µm. (**f**) Non-permeabilized cells were immunostained with an anti-FLT3 extracellular domain (ECD) antibody. Bars, 10 µm. Note that FLT3 tyrosine kinase inhibitors inactivated FLT3, then released the receptor from the Golgi region for localization to the PM.

Recently, we reported that constitutively active KIT mutants in MCL, GIST, or AML accumulate in organelles in a manner dependent on their tyrosine kinase activity^21,22,24^. Thus, we asked whether FLT3-ITD tyrosine kinase activity was required for retention of the mutant in the Golgi region. To answer this, we treated AML cells with quizartinib or midostaurin, small molecule TKIs (hereafter, referred to as AC220 and PKC412, respectively), which block the activation of FLT3^7,8,14,28,31,32^. Treatment of MOLM-14 cells with the TKIs suppressed the autophosphorylation of FLT3 at Tyr842 (pFLT3^Y842^) and pFLT3^Y591^ within 4 h, resulting in a decrease in phospho-AKT (pAKT), pERK, and pSTAT5 (Fig. 3a,b; Suppl. Fig. 3a). Treatment of MV4-11/Kasumi-6 with the PKC412 gave similar results (Suppl. Fig. 3b,c), confirming that the activation of AKT, ERK, and STAT5 is dependent on the FLT3-ITD activity. As shown in Fig. 3c, these TKIs suppressed the proliferation of MOLM-14. In our immunoblot analyses, FLT3 was observed as doublet bands (Fig. 3a,b, at about 150 kDa and 130 kDa). Peptide *N*-glycosidase F (PNGase F), which digests all N-linked glycosylation, moved FLT3 bands to the position of its deglycosylated (DG) form (Fig. 3d; Suppl. Fig. 3d). Endoglycosidase H (Endo H) removes high mannose (HM) but not complex glycosylation (CG). The lower band of FLT3 (130 kDa) disappeared in the presence of Endo H, whereas the upper band of FLT3 (150 kDa) was not affected by the enzyme (Fig. 3d; Suppl. Fig. 3d). Therefore, the upper and lower bands of FLT3 consisted of the CG and HM form, respectively, similar to KIT^21,22,33,34^.

To check the effect of AC220 or PKC412 on FLT3 localization, we immunostained permeabilized MOLM-14 cells with an anti-FLT3 antibody. Interestingly, TKI treatment markedly decreased the FLT3 level in the Golgi region (Fig. 3e). Conversely, we found that the treatment increased the level of FLT3, probably within the PM region (Fig. 3e, see insets). Previous reports showed that a kinase-dead mutation of FLT3-ITD or TKIs (AC220/crenolanib) enhance PM distribution of the mutant receptors^15,17,35,36^. Therefore, we examined the PM levels of FLT3-ITD on non-permeabilized MOLM-14 cells by staining for the FLT3 extracellular domain (ECD). As shown in Fig. 3f, treatment with AC220 or PKC412 enhanced the PM staining of FLT3, similar to previous reports on crenolanib-treated MV4-11^35^. Taken together, these results suggest that FLT3-ITD remains in the Golgi region during secretory trafficking in a manner dependent on its kinase activity and that TKIs move the mutant receptor to the PM.

### In AML cells, FLT3-ITD can activate STAT5, ATK, and ERK in early secretory compartments

**Figure 4:**
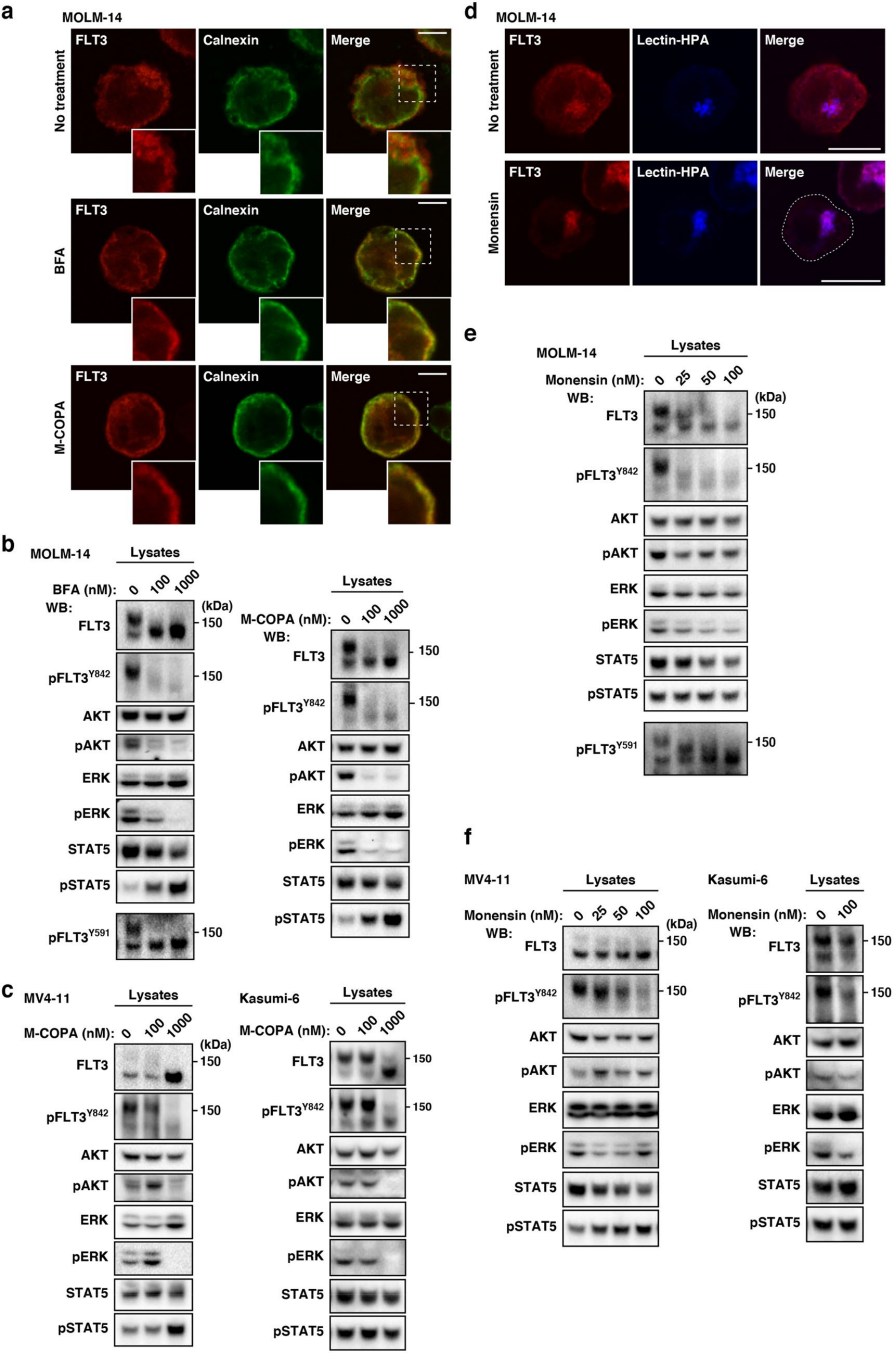
In AML cells, FLT3-ITD can activate AKT, ERK, and STAT5 before it reaches the PM. (**a**–**c**) MOLM-14 (**a**, **b**), MV4-11, or Kasumi-6 cells (**c**) were treated with inhibitors of ER export (BFA or M-COPA) for 8 h. (**a**) MOLM-14 cells treated with 1 µM BFA (middle panels) or 1 µM M-COPA (bottom panels) were stained with anti-FLT3 (red) and calnexin (ER marker, green). Insets show the magnified images of the boxed area. Bars, 10 µm. (**b**) Lysates were immunoblotted for FLT3, phospho-FLT3 Tyr842 (pFLT3^Y842^), AKT, pAKT, ERK, pERK, STAT5, and pSTAT5. To examine pFLT3^Y591^, FLT3 was immunoprecipitated, then immunoblotted. (**c**) MV4-11 (left) or Kasumi-6 cells (right) were treated with M-COPA for 8 h, then immunoblotted. Full length blots are presented in Supplementary Fig. 5. Note that BFA and M-COPA inhibited the activation of AKT and ERK but not that of STAT5 through blocking FLT3-ITD trafficking from the ER to the Golgi apparatus. (**d**, **e**) MOLM-14 cells were treated with monensin (inhibitor of Golgi export) for 8 h. (**d**) Cells treated with 100 nM monensin were stained with anti-FLT3 (red) and lectin-HPA (Golgi marker, blue). Dashed line, cell border. Bars, 10 µm. (**e**) Lysates were immunoblotted with the indicated antibody. To examine pFLT3^Y591^, FLT3 was immunoprecipitated, then immunoblotted. Full length blots are presented in Supplementary Fig. 5. (**f**) MV4-11 (left) or Kasumi-6 cells (right) were treated with monensin for 8 h, then immunoblotted. Full length blots are presented in Supplementary Fig. 6.

Finally, we examined the relationship between FLT3-ITD localization and growth signals. To determine whether FLT3-ITD activated downstream molecules before reaching the PM, we treated AML cells with BFA, M-COPA (blockers of ER export to the Golgi^24–26,37^), or monensin (an inhibitor of secretory trafficking thorough blocking Golgi export^21,22,33,38,39^). Previous studies showed that BFA, an inhibitor of ER export^37^, suppresses the activation of AKT and ERK but not STAT5 in MV4-11 cells^4,40^. Recently, we reported that in addition to BFA, M-COPA blocks trafficking of KIT mutants from the ER^24–26^. Thus, we treated AML cells with M-COPA as well as BFA to confirm the effect of blockade of ER export on FLT3 signaling. Our immunofluorescence assay on MOLM-14 cells showed that BFA/M-COPA treatment decreased FLT3 levels in the Golgi region within 8 h and greatly increased the co-localization of calnexin (ER marker) with FLT3 (Fig. 4a, see inset panels), confirming that these inhibitors block biosynthetic protein transport from the ER to the Golgi apparatus. Next, we performed immunoblotting. As shown in Fig. 4b, in the presence of BFA/M-COPA, FLT3-ITD was retained in a high mannose form (see upper panels), confirming blockade of complex glycosylation in the Golgi apparatus. Consistent with a previous report^4^, pFLT3^Y842^ decreased with BFA/M-COPA treatment, indicating that the phosphorylation does not occur in the ER. On the other hand, pFLT3^Y591^ was maintained in the ER (Fig. 4b, left, bottom panel). In MV4-11 and Kasumi-6 as well as MOLM-14, FLT3-ITD in the ER was unable to activate AKT and ERK (Fig. 4c; Suppl. Fig. 4a,b). Blockade of ER export, however, did not inhibit STAT5 activation through FLT3-ITD (Fig. 4b,c; Suppl. Fig. 4a,b). These results indicate that ER-retained FLT3-ITD activates STAT5 but not AKT and ERK.

Next, we asked whether FLT3-ITD in the Golgi apparatus can activate AKT or ERK by using monensin, a blocker of Golgi export^21,22,33,38,39^. As shown in Fig. 4d, our immunofluorescence assay showed that FLT3 distribution other than in the Golgi region was markedly decreased in MOLM-14 cells in the presence of 100 nM monensin for 8 h, confirming the expectation that the treatment blocks Golgi export of FLT3-ITD. Only the high-mannose form of the FLT3 bands was found in the presence of monensin (Fig. 4e, top panel). In the presence of monensin, pFLT3^Y842^ was decreased (Fig. 4e), whereas pFLT3^Y591^ remained unchanged (Fig. 4e, bottom panel), suggesting that the treatment does not block all tyrosine phosphorylations in FLT3-ITD and that these tyrosine residues in FLT3 are regulated differently. Blocking the PM localization of FLT3-ITD with monensin in MOLM-14 caused it to be partially decreased in pAKT and pERK. Although monensin lowered the protein level of STAT5, pSTAT5 remained. We found FLT3 signals in the presence of monensin for 24 h (Suppl. Fig. 4c). The results obtained with this cell line indicate that the activation of AKT and ERK via FLT3-ITD occurs not only at the PM but also in the Golgi and support our finding that STAT5 is activated before FLT3-ITD moves to the PM. Although blocking Golgi export of FLT3 with monensin altered levels of pAKT, pERK, and STAT5, these effects were various among MOLM-14, MV4-11, and Kasumi-6 (Fig. 4e,f), and the persistence of this phosphorylation in the presence of monensin was common to all these AML cell lines. These results indicate that FLT3-ITD can activate downstream before it reaches the PM. Taken together, these results suggest that in AML cells, FLT3-ITD can activate STAT5 and AKT/ERK on the ER and the Golgi apparatus, respectively.

## Discussion

**Figure 5:**
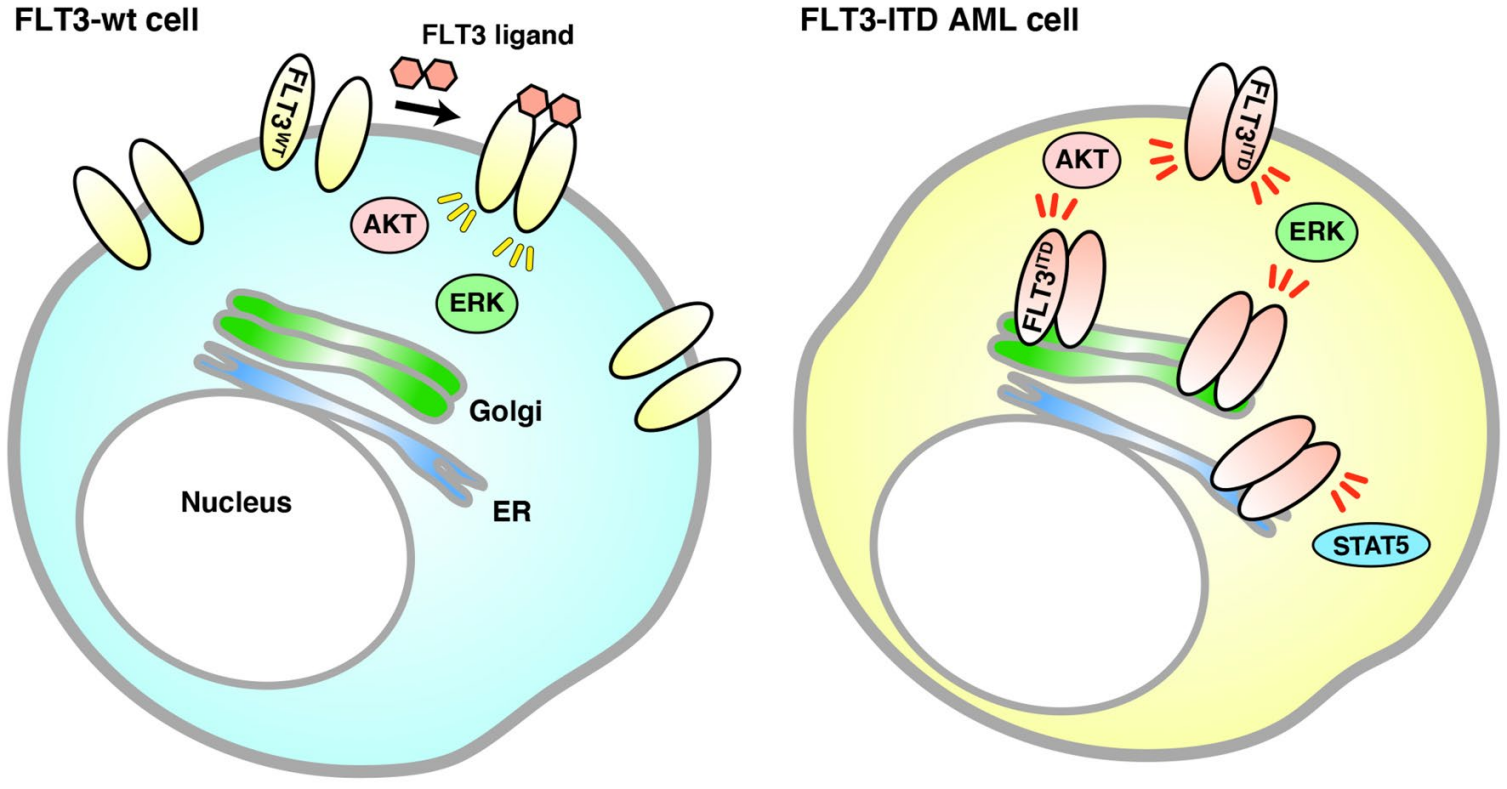
Model of FLT3-ITD signaling on intracellular compartments in AML cells. (Left) FLT3-wt normally moves to the PM along the secretory pathway for binding its ligand. Upon stimulation with FLT3 ligand at the cell surface, the wild-type receptor activates downstream molecules. (Right) FLT3-ITD is retained in the Golgi apparatus in AML cells. The mutant can activate kinases, such as AKT and ERK, in the perinuclear Golgi region, but not in the ER before reaching the PM. On the other hand, FLT3-ITD activates STAT5 in the ER, where it is newly synthesized.

In this study, we demonstrated that unlike FLT3-wt (Fig. 5, left), FLT3-ITD accumulates in early secretory organelles, such as the Golgi apparatus, and in that location, causes tyrosine phosphorylation signaling in leukemia cells (Fig. 5, right). The Golgi retention of FLT3-ITD is dependent on the tyrosine kinase activity of the mutant. TKI increases PM levels of FLT3-ITD by releasing the mutant from the Golgi apparatus. FLT3-ITD in the Golgi region can activate AKT and ERK, whereas that in the ER triggers STAT5 phosphorylation, leading to autonomous cell proliferation.

Recently, we reported that in MCL, KIT^D816V^ (human) or KIT^D814Y^ (mouse) activates STAT5 and AKT on the ER and endolysosomes, respectively^21,25^, whereas KIT^V560G^ in MCL activates them at the Golgi apparatus^24^. Furthermore, KIT mutants including KIT^D816V^ in cells other than MCL, such as GIST and blood cells, cause oncogenic signals on the Golgi apparatus^22,24,33^. As previously described^4,18,19^, we confirmed that after biosynthesis in the ER, FLT3-ITD causes STAT5 tyrosine phosphorylation in a manner similar to that of KIT^D816V^ in MCL. On the other hand, activation of AKT and ERK through FLT3-ITD is similar to activation through the KIT mutant in GIST in that it occurs on the Golgi apparatus. A recent report showed that FLT3^D835Y^ is also found in endomembranes^41^. As described above, since the signal platform for a kinase may be affected by its mutation site, there is great interest in carrying out a further investigation to determine whether FLT3^D835Y^ causes growth signaling on the ER, Golgi, or endosome/lysosomes.

Recently, novel protein interactions and downstream molecules for FLT3 which support cancer cell proliferation have been identified. One FLT3 mutant was found to activate Rho kinase through activation of RhoA small GTPase, resulting in myeloproliferative disease development^42^. GADS physically associates with FLT3-ITD, and the interaction enhances downstream activation^12^. Analysis of spatio-temporal associations of FLT3 mutants with these functional interactors is an attractive possibility.

Previous studies showed that other RTK mutants, such as FGFR3^K650E^ in multiple myeloma, RET multiple endocrine neoplasia type 2B (RET^MEN2B^), and PDGFRA^Y289C^, are also tyrosine-phosphorylated via a secretory pathway^39,43–49^. Signal transduction from the secretory compartments may be a characteristic feature of a large number of RTK mutants. Early secretory compartments can be subdivided into the ER, the ER-Golgi intermediate compartment, *cis*-, *medial*-Golgi cisternae, TGN, and others. It would be interesting to identify the sub-compartment at which RTK mutants are retained for precise understanding of the mechanism of growth signaling. Three-dimensional super-resolution confocal microscopic analysis of cancer cells is now under way.

Golgi retention of FLT3-ITD is dependent on receptor tyrosine kinase activity. As with previous reports^17,36^, we confirmed that a TKI increased PM localization of FLT3-ITD, indicating that these inhibitors can release the mutant from the Golgi region for localization to the PM. Other reports together with the results of our studies showed that TKIs increase the PM levels of RTK mutants, such as EGFR(T790M), KIT(D816V), and PDGFRA(V561G)^21,24,50–53^. Enhancement of PM distribution with TKIs may be a common feature of RTK mutants. Furthermore, recent studies showed that the effect of chimeric antigen receptor T-cell therapy and antigen-dependent cell cytotoxicity using anti-FLT3 is enhanced by increasing the PM levels of FLT3-ITD through TKI treatment^30,35,36,53^. Combining TKIs together with immunotherapy will lead to improvements in the prognosis of cancer patients.

TKIs and antibodies against RTKs have been developed for suppression of growth signals in cancers. In this study, blockade of the ER export of FLT3-ITD with BFA/M-COPA greatly reduced tyrosine phosphorylation signals in AML cells. Since the bioavailability of M-COPA in vivo is higher than that of BFA and because it can be orally administered to animals, we will investigate the anti-cancer effect of the compound on AML-bearing mice. Together with the results of previous reports^24–26,54–58^, our findings suggest that an intracellular trafficking blockade of RTK mutants could be a third strategy for inhibition of oncogenic signaling.

In conclusion, we showed that in AML cells, the perinuclear region where FLT3-ITD accumulates is the Golgi apparatus. Similar to KIT mutants in GISTs, FLT3-ITD is retained at the Golgi region in a manner dependent on its kinase activity, but TKI releases the mutant to the PM. Our findings provide new insights into the role of FLT3-ITD in autonomous AML cell growth. Moreover, from a clinical point of view, our findings offer a new strategy for AML treatment through blocking the involvement of FLT3-ITD in secretory trafficking.

## Materials and methods

### Cell culture

RS4-11, MV4-11, THP-1 (American Type Culture Collection, Manassas, VA), and MOLM-14 (Leibniz Institute DSMZ-German Collection of Microorganisms and Cell Cultures GmbH, Braunschweig, Germany) were cultured at 37 °C in RPMI1640 medium supplemented with 10% fetal calf serum (FCS), penicillin/streptomycin, glutamine (Pen/Strep/Gln), and 50 µM 2-mercaptoethanol (2-ME). Kasumi-6 cells (Japanese Collection of Research Bioresources Cell Bank, Osaka, Japan) were cultured at 37 °C in RPMI1640 medium supplemented with 20% FCS, 2 ng/mL granulocyte–macrophage colony-stimulating factor (Peprotech, Rocky Hill, NJ), Pen/Strept/Gln, and 50 µM 2-ME. All human cell lines were authenticated by Short Tandem Repeat analysis and tested for *Mycoplasma* contamination with a MycoAlert Mycoplasma Detection Kit (Lonza, Basel, Switzerland).

### Chemicals

AC220 and PKC412 (Selleck, Houston, TX) were dissolved in dimethyl sulfoxide (DMSO). BFA (Sigma-Aldrich, St. Louis, MO) and monensin (Biomol, Hamburg, Germany) were dissolved in ethanol or methanol, respectively. M-COPA (also known as AMF-26) was synthesized as previously described^59,60^ and dissolved in DMSO.

### Antibodies

The sources of purchased antibodies were as follows: FLT3 (S-18), FLT3 (SF1.340), ERK (K-23), and STAT5 (C-17) from Santa Cruz Biotechnology (Dallas, TX); FLT3 (8F2), FLT3[pY842] (10A8), FLT3[pY591] (54H1), AKT (40D4), AKT[pT308] (C31E5E), STAT5 (D2O6Y), STAT5[pY694] (D47E7), ERK1/2 (137F5), and ERK[pT202/pY204] (E10) from Cell Signaling Technology (Danvers, MA); TfR (ab84036), TGN46 (ab76282), and GM130 (EP892Y) from Abcam (Cambridge, UK); STAT5 (89) from BD Transduction Laboratories (Franklin Lakes, NJ); Calnexin (ADI-SPA-860) from Enzo (Farmingdale, NY); LAMP1 (L1418) from Sigma (St. Louis, MO) and FLT3 (MAB812) from R&D Systems (Minneapolis, MN). Horseradish peroxidase-labeled (HRP-labeled) donkey anti-mouse IgG and anti-rabbit IgG secondary antibodies were purchased from The Jackson Laboratory (Bar Harbor, MA). Alexa Fluor-conjugated (AF-conjugated) donkey secondary antibodies were obtained from Thermo Fisher Scientific (Rockford, IL). The list of antibodies with sources and conditions of immunoblotting and immunofluorescence is shown in Suppl. Table 1.

### Immunofluorescence confocal microscopy

Leukemia cells in suspension culture were fixed with 4% paraformaldehyde (PFA) for 20 min at room temperature, then cyto-centrifuged onto coverslips. Fixed cells were permeabilized and blocked for 30 min in phosphate-buffered saline (PBS) supplemented with 0.1% saponin and 3% bovine serum albumin (BSA), and then incubated with a primary and a secondary antibody for 1 h each. AF647-conjugated lectin*-Helix pomatia* agglutinin (lectin-HPA, Thermo Fisher Scientific) was used for Golgi staining. After washing with PBS, cells were mounted with Fluoromount (DiagnosticBioSystems, Pleasanton, CA). For staining the extracellular domain of FLT3, living MOLM-14 cells were stained with anti-FLT3 (SF1.340) and AF488-conjugated anti-mouse IgG in PBS supplemented with 3% BSA and 0.1% sodium azide (NaN_3_) at 4 °C for 1 h each. Stained cells were fixed with 4% PFA for 20 min at room temperature. Confocal images were obtained with an Fluoview FV10i (Olympus, Tokyo, Japan) or a TCS SP5 II/SP8 (Leica, Wetzlar, Germany) laser scanning microscope. Composite figures were prepared with an FV1000 Viewer (Olympus), LAS X (Leica), Photoshop, and Illustrator software (Adobe, San Jose, CA).

### Western blotting

Lysates prepared in SDS-PAGE sample buffer were subjected to SDS-PAGE and electro-transferred onto PVDF membranes. Basically, 5% skimmed milk in tris-buffered saline with Tween 20 (TBS-T) was used for diluting antibodies. For immunoblotting with anti-FLT3[pY842] (10A8) or anti-FLT3[pY591] (54H1), the antibody was diluted with 3% BSA in TBS-T. Immunodetection was performed with Enhanced Chemiluminescence Prime (PerkinElmer, Waltham, MA). Sequential re-probing of membranes was performed after the complete removal of primary and secondary antibodies in stripping buffer (Thermo Fisher Scientific), or inactivation of HRP by 0.1% NaN_3_. Results were analyzed with an LAS-3000 with Science Lab software (Fujifilm, Tokyo, Japan) or a ChemiDoc XRC + with Image Lab software (BIORAD, Hercules, CA). Full length blots are presented in Suppl. Figs. 5 and 6.

### Immunoprecipitation

Lysates from 1 to 5 × 10^6^ cells were prepared in NP-40 lysis buffer (50 mM HEPES, pH 7.4, 10% glycerol, 1% NP-40, 4 mM EDTA, 100 mM NaF, 1 mM Na_3_VO_4_, cOmplete™ protease inhibitor cocktail (Sigma), and 1 mM PMSF). Immunoprecipitation was performed at 4 °C for 3–5 h using protein G Sepharose (GE Healthcare Life Sciences, Uppsala, Sweden) or Dynabeads (Thermo Fisher Scientific) pre-coated with anti-FLT3 (S-18). Immunoprecipitates were dissolved in SDS-PAGE sample buffer.

### Cell proliferation assay

Cells were cultured with PKC412 or AC220 for 48 h. Cell proliferation was quantified using the CellTiter-GLO Luminescent Cell Viability Assay (Promega, Madison, WI), according to the manufacturer’s instructions. ATP production was measured with an ARVO X3 2030 Multilabel Reader (PerkinElmer, Waltham, MA).

### Analysis of protein glycosylation

Following the manufacturer’s instructions (New England Biolabs, Ipswich, MA), NP-40 cell lysates were treated with endoglycosidases for 1 h at 37 °C. Since the FLT3 expression level in THP-1 was low for this assay, FLT3 was concentrated by immunoprecipitation with anti-FLT3 (S-18), and then treated with endoglycosidases. The reactions were stopped with SDS-PAGE sample buffer, and products were resolved by SDS-PAGE and immunoblotted.

## Supplementary Information

Supplementary Information.

## Data Availability

All datasets used and/or analyzed during the current study are available from the corresponding author upon reasonable request.
