# Supplementary material for: FLT3-ITD transduces autonomous growth signals during its biosynthetic trafficking in acute myelogenous leukemia cells
Source: Sci Rep. 2021 Nov 22;11:22678. doi: 10.1038/s41598-021-02221-2 (PMC8608843; doi:10.1038/s41598-021-02221-2)
Supplement: Supplementary file 1 — Supplementary Information. [file 41598_2021_2221_MOESM1_ESM.pdf]

Supplementary Figures

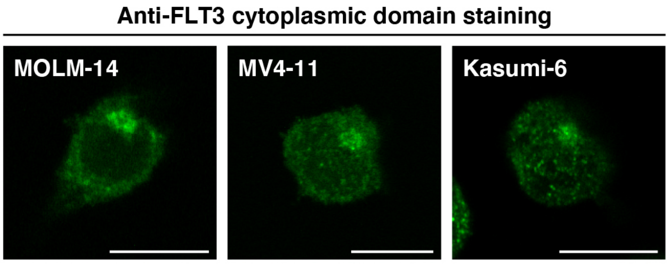

Supplementary Figure 1. An anti-FLT3 cytoplasmic domain antibody stains the perinuclear region of MOLM-14, MV4-11, and Kasumi-6 cells. *FLT3-ITD*-harboring AML cell lines were immunostained with an anti-FLT3 cytoplasmic domain antibody. Bars, 10 μm.

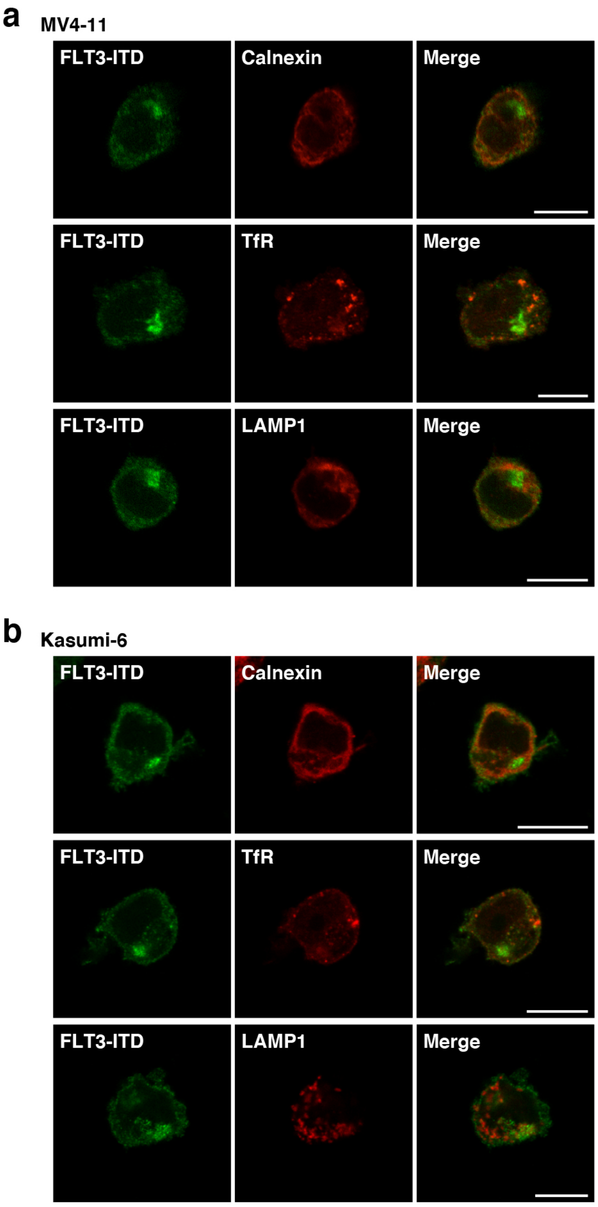

Supplementary Figure 2. In MV4-11 and Kasumi-6 cells, the perinuclear region, where FLT3-ITD localized, is not ER, endosomes, or lysosomes. (**a**,**b**) MV4-11 (**a**) or Kasumi-6 cells (**b**) were immunostained for FLT3 (green) in conjunction with the indicated organelle markers (red). Calnexin (ER marker); TfR (endosome marker); LAMP1 (lysosome marker). Bars, 10 µm.

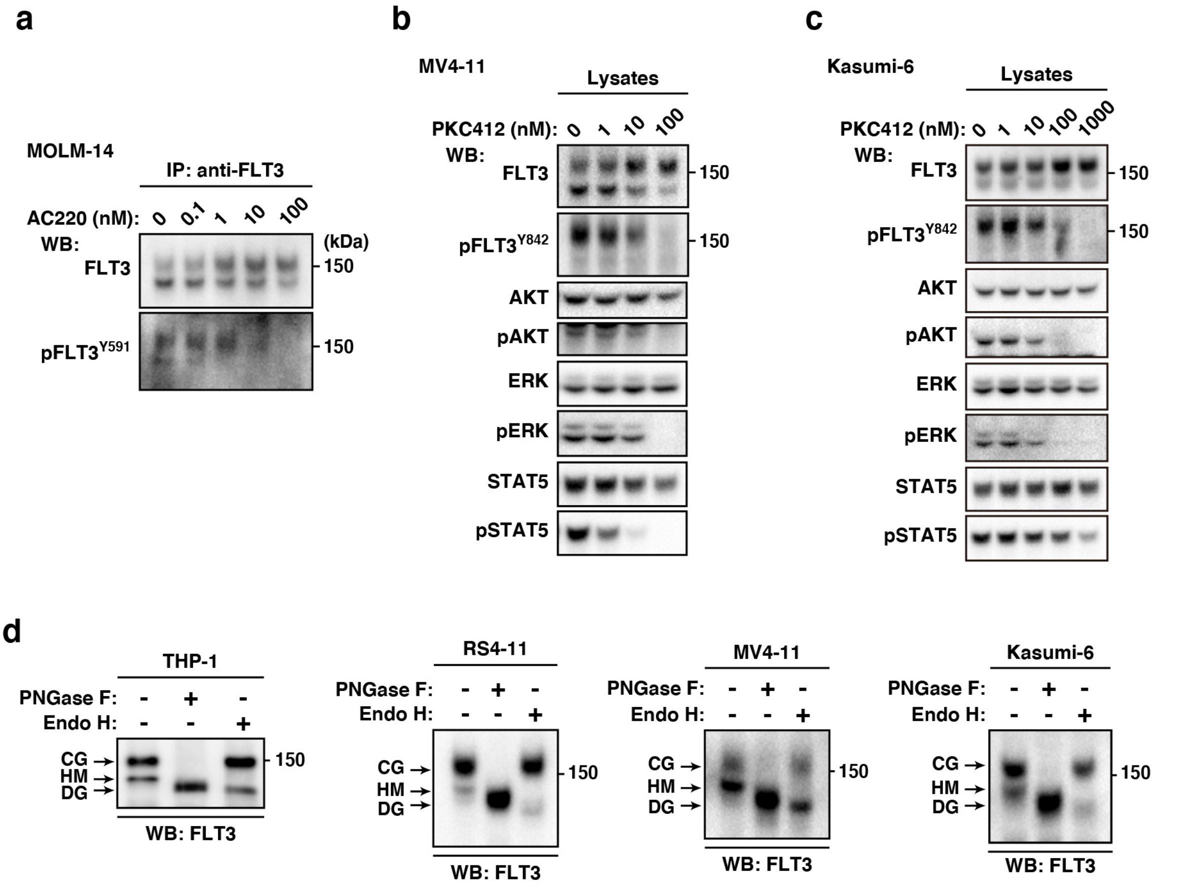

Supplementary Figure 3. Tyrosine kinase inhibitors block the phosphorylation of AKT, ERK, and STAT5 through suppressing FLT3-ITD activation. (**a**) MOLM-14 cells were treated with AC220 for 4 hours. FLT3 were immunoprecipitated with anti-FLT3 antibody, then immunoblotted for FLT3 and phospho-FLT3 Tyr591 (pFLT3^{Y591}). (**b**,**c**) MV4-11 (**b**) or Kasumi-6 cells (**c**) were treated for 4 hours with PKC412 (inhibitor of FLT3 tyrosine kinase). Lysates were immunoblotted for FLT3, phospho-FLT3 Tyr842 (pFLT3^{Y842}), AKT, pAKT, ERK, pERK, STAT5, and pSTAT5. (**d**) FLT3 immunoprecipitates (THP-1) or lysates (RS4-11, MV4-11, and Kasumi-6) were treated with peptide N-glycosidase F (PNGase F) or endoglycosidase H (endo H) then immunoblotted with anti-FLT3. CG, complex-glycosylated form; HM, high mannose form; DG, deglycosylated form. Full length blots are presented in Supplementary Figure 6.

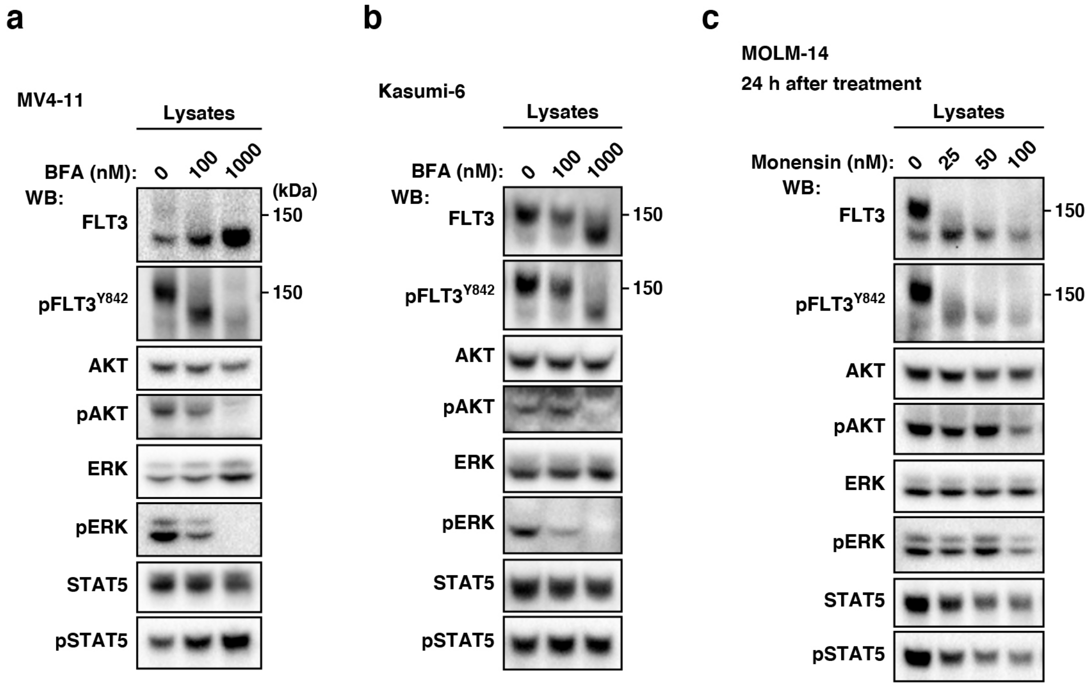

Supplementary Figure 4. In AML cells, FLT3-ITD can activate AKT, ERK, and STAT5 before it reaches the PM. (**a**,**b**) MV4-11 (**a**) or Kasumi-6 cells (**b**) were treated for 8 hours with brefeldin A (BFA, inhibitor of ER export) for 8 hours. Lysates were immunoblotted for FLT3, phospho-FLT3 Tyr842 ($pFLT3^{Y842}$), AKT, pAKT, ERK, pERK, STAT5, and pSTAT5. (**c**) MOLM-14 cells were treated with monensin (inhibitor of Golgi export) for 24 hours. Lysates were immunoblotted. Full length blots are presented in Supplementary Figure 6.

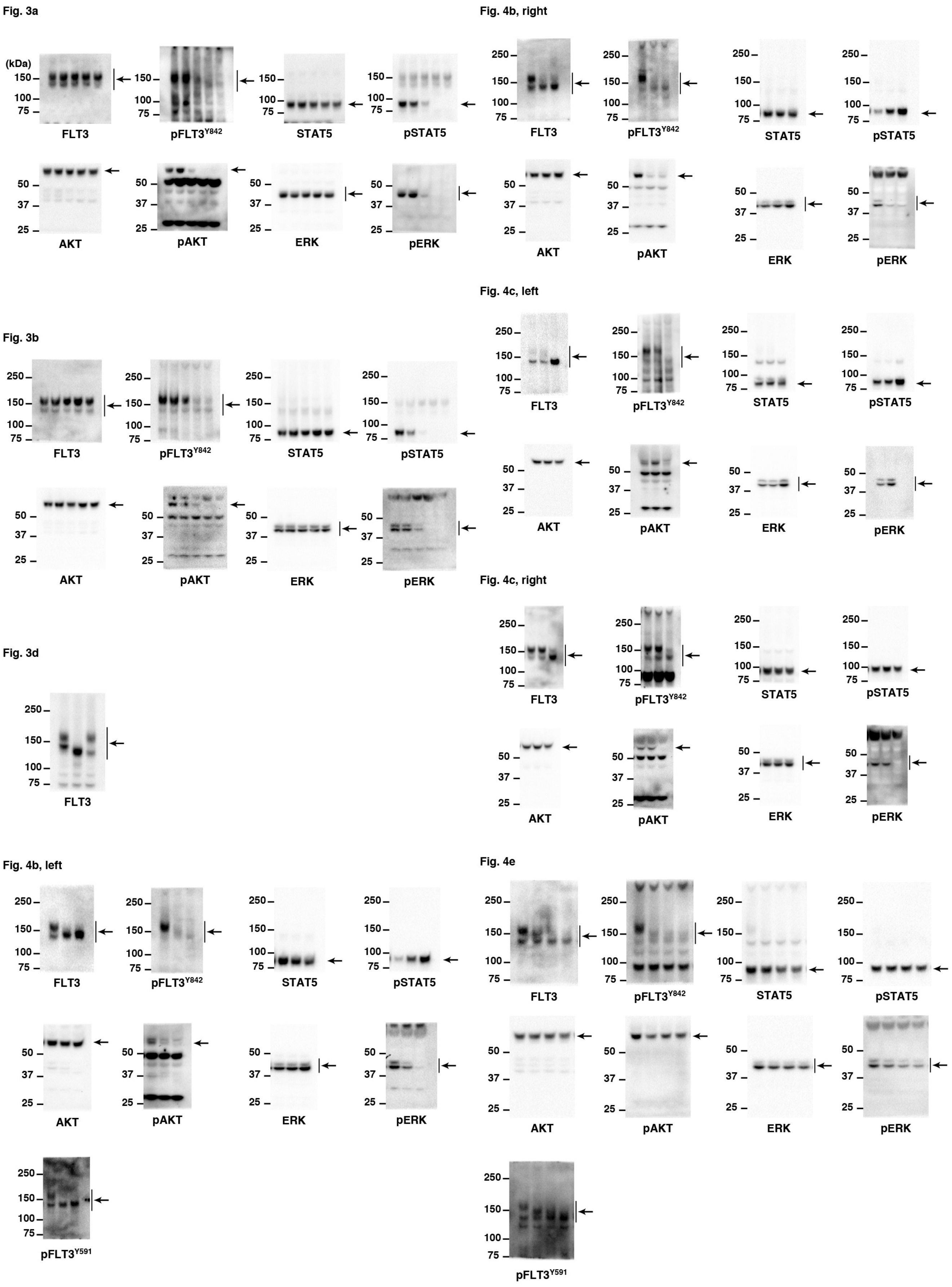

Supplementary Figure 5. Uncropped versions of the immunoblots of Figures 3a-4e.

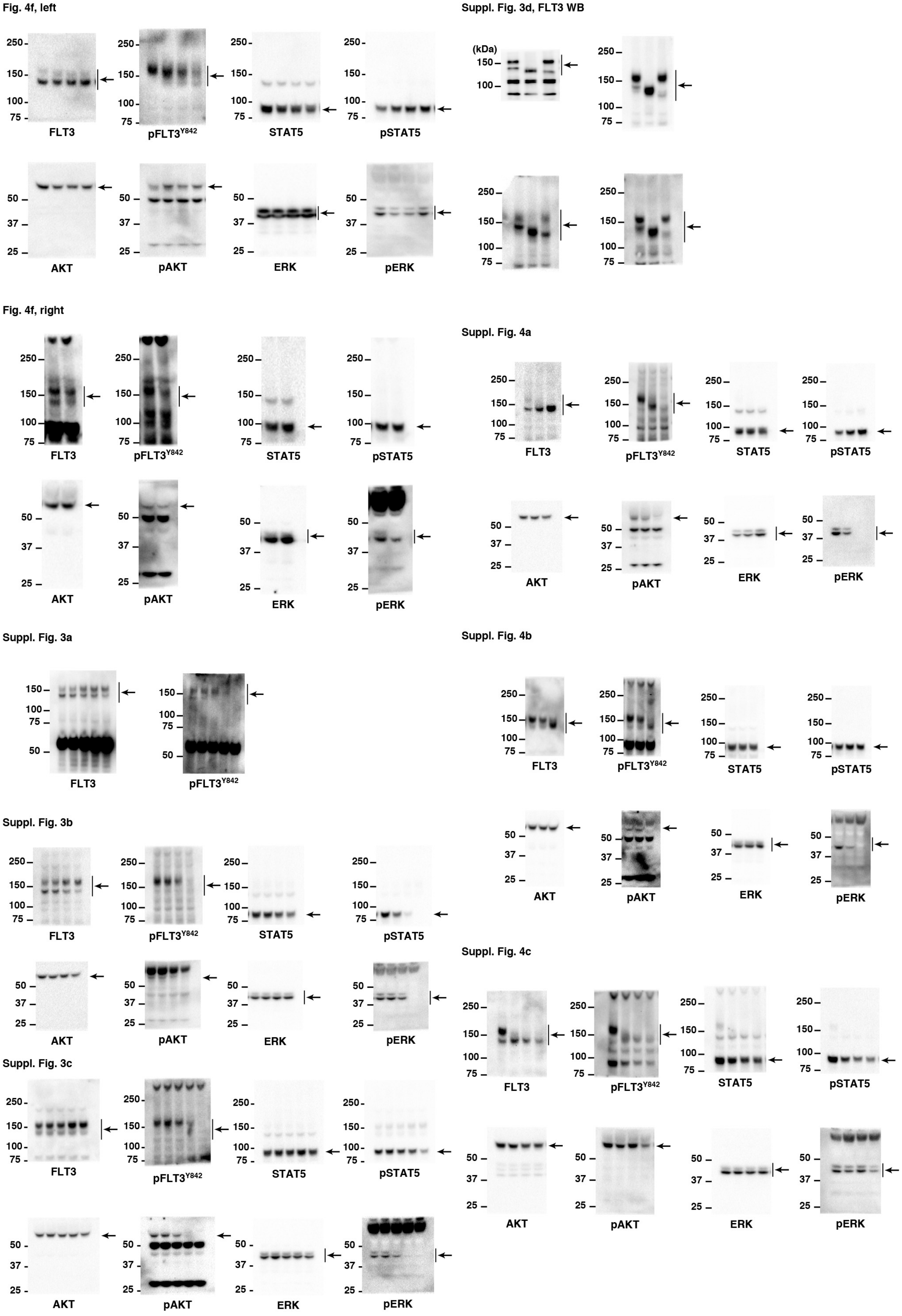

Supplementary Figure 6. Uncropped versions of the immunoblots of Figure 4f and Supplementary Figures 3a-4c.

Supplementary Table 1. List of antibodies

Antibody	Clone/catalog #	Distribution source	WB	IF
AKT	40D4	Cell Signaling Technology	1/1000	-
AKT [pT308]	C31E5E	Cell Signaling Technology	1/1000	-
Calnexin	ADI-SPA-860	Enzo	-	1/250
FLT3	S-18	Santa Cruz Biotechnology	1/1000	-
FLT3	8F2	Cell Signaling Technology	1/1000	1/250
FLT3	MAB812	R&D Systems	-	1/50~1/250
FLT3	SF1.340	Santa Cruz Biotechnology	-	1/100~1/250
ERK2	K-23	Santa Cruz Biotechnology	1/1000~1/2000	-
ERK1/2	137F5	Cell Signaling Technology	1/1000~1/2000	-
ERK [pT202/pY204]	E10	Cell Signaling Technology	1/1000	-
FLT3 [pY842]	10A8	Cell Signaling Technology	1/1000	-
FLT3 [pY591]	54H1	Cell Signaling Technology	1/1000	-
GM130	EP892Y	Abcam	-	1/250
LAMP1	L1418	Sigma-Aldrich	-	1/250
STAT5	C-17	Santa Cruz Biotechnology	1/1000	-
STAT5	89	BD Transduction Laboratories	1/1000	-
STAT5	D2O6Y	Cell Signaling Technology	1/1000	-
STAT5 [pY694]	D47E7	Cell Signaling Technology	1/1000	-
TfR	ab84036	Abcam	-	1/250
TGN46	ab76282	Abcam	-	1/100
HRP donkey anti-mouse IgG	715-035-151	Jackson Laboratory	1/2000	-
HRP donkey anti-rabbit IgG	711-035-152	Jackson Laboratory	1/2000	-
AF488 donkey anti-mouse IgG	A21202	Thermo Fisher Scientific	-	1/250
AF488 donkey anti-rabbit IgG	A21206	Thermo Fisher Scientific	-	1/250
AF568 donkey anti-mouse IgG	A10037	Thermo Fisher Scientific	-	1/250
AF568 donkey anti-rabbit IgG	A10042	Thermo Fisher Scientific	-	1/250
AF647 lectin-HPA	L32454	Thermo Fisher Scientific	-	1/100~1/250

Supplementary Table 1. List of antibodies. The list shows antibodies with sources and conditions of Western blotting (WB) and immunofluorescence (IF).
